# IMPACT OF COVID-19 PANDEMIC ON MENTAL HEALTH IN OLDER ADULTS: COMPARISON BETWEEN 2020 AND 2022

**DOI:** 10.1093/geroni/igac059.2980

**Published:** 2022-12-20

**Authors:** Dara Kiu, Yi Leung, Tianyin Liu, Wen Zhang, Wai-wai Kwok, Lesley Sze, Gloria H Y Wong, Terry Lum

**Affiliations:** The University of Hong Kong, Hong Kong, Hong Kong; The University of Hong Kong, Hong Kong, Hong Kong; The University of Hong Kong, Hong Kong, Hong Kong; The University of Hong Kong, Hong Kong, Hong Kong; The University of Hong Kong, Hong Kong, Hong Kong; The University of Hong Kong, Hong Kong, Hong Kong; The University of Hong Kong, Hong Kong, Hong Kong; The University of Hong Kong, Hong Kong, Hong Kong

## Abstract

The COVID-19 pandemic had a major impact on older adults’ mental health, but less is known about its longer-term effect. We examined changes in depressive and anxiety symptoms among older adults between the onset and two years into the pandemic. Data were drawn from two cross-sectional telephone surveys conducted with older adults aged ≥ 60 years in Hong Kong in 2020 and 2022. Respondents were screened for depression and anxiety using Patient Health Questionnaire-2 (PHQ-2) and General Anxiety Disorders-2 (GAD-2) and, if screened positive (i.e. scoring ≥ 3 in PHQ-2 or GAD-2), evaluated with PHQ-9 and GAD-7 for symptom severity. After case-control matching baseline age, gender, living districts, and pre-existing mental health conditions based on the respondent ratio between the two surveys (i.e. 2:1 ratio), 4095 and 2099 respondents from the 2020 and 2022 surveys were included in the analysis. Respondents’ average baseline age was 75 years old, 77% were female, and 13% had a pre-existing mental health condition. There were significant increases in the proportion of older adults screened positive for depression (8.3% to 13.5%) and anxiety (6.9% to 11.4%) and a significant increase in depressive symptom severity (4.63 to 7.72) between 2020 and 2022 (p < .001). Logistic regression suggested that, over two years, older adults with pre-existing mental health conditions were 1.59 times more likely to screen positive for depression than those without such conditions. Linear regression suggested that males were associated with increased depressive (B=-2.42, p=.004) and anxiety (B=-2.49, p=.021) symptom severity than females over the years.

